# MicroRNA-101 targets EZH2, MCL-1 and FOS to suppress proliferation, invasion and stem cell-like phenotype of aggressive endometrial cancer cells

**DOI:** 10.18632/oncotarget.2157

**Published:** 2014-07-02

**Authors:** Yosuke Konno, Peixin Dong, Ying Xiong, Fumihiko Suzuki, Jiabin Lu, Muyan Cai, Hidemichi Watari, Takashi Mitamura, Masayoshi Hosaka, Sharon J.B. Hanley, Masataka Kudo, Noriaki Sakuragi

**Affiliations:** ^1^ Department of Gynecology, Hokkaido University, Sapporo, Japan; ^2^ Department of Women's Health Educational System, Hokkaido University, Sapporo, Japan; ^3^ Department of Gynecology, State Key Laboratory of Oncology in South China, Sun Yat-sen University Cancer Center, Guangzhou, P. R. China; ^4^ Department of Obstetrics and Gynecology, Tohoku University, Sendai, Japan; ^5^ Department of Pathology, State Key Laboratory of Oncology in South China, Sun Yat-sen University Cancer Center, Guangzhou, P. R. China

**Keywords:** microRNA-101, proliferation, EMT, EZH2, MCL-1, FOS

## Abstract

MicroRNA-101 has been implicated as a tumor suppressor miRNA in human tumors. However, its potential functional impact and the underlying mechanisms in endometrial cancer progression have not been determined. Here, we report that in aggressive endometrial cancer cells, re-expression of microRNA-101 leads to inhibition of cell proliferation and induction of apoptosis and senescence. Ectopic overexpression of microRNA-101 attenuates the epithelial-mesenchymal transition-associated cancer cell migration and invasion, abrogates the sphere-forming capacity and enhances chemosensitivity to paclitaxel. Algorithm and microarray-based strategies identifies potential microRNA-101 targets. Among these, we validated *EZH2*, *MCL-1* and *FOS* as direct targets of miR-101 and silencing of these genes mimics the tumor suppressive effects observed on promoting microRNA-101 function. Importantly, further results suggest an inverse correlation between low miR-101 and high EZH2, MCL-1 and FOS expression in EC specimens. We conclude that, as a crucial tumor suppressor, microRNA-101 suppresses cell proliferation, invasiveness and self-renewal in aggressive endometrial cancer cells via modulating multiple critical oncogenes. The microRNA-101-EZH2/MCL-1/FOS axis is a potential therapeutic target for endometrial cancer.

## INTRODUCTION

MicroRNAs (miRs) are naturally occurring, short, noncoding RNAs that repress the expression of multiple protein-coding mRNAs by repressing translation and/or by causing mRNA degradation [1]. The miRNAs can regulate a wide range of cellular processes including proliferation, apoptosis, senescence, differentiation and development [2, 3]. Dysregulation of miRNAs contributes to tumorigenesis and metastasis [4, 5]. Several miRNAs are restricted to certain types of tumors, indicating their tissue-specific functions in cancer [6]. However, other miRNAs (such as miR-101 and miR-145) are found in various human tumors and predicted to have broad biological functions [7, 8]. In particular, miR-101 can play a tumor-suppressive role in regulating tumor cell growth, migration, invasion, drug resistance and cancer stem cell (CSC) characteristics via suppression of oncogenic signaling pathways in breast [9], colon [10], lung [11], ovarian [12] and gastric cancers [13].

In contrast to well-differentiated, endometrioid endometrial cancers (ECs) with a good prognosis, poorly-differentiated endometrioid and serous ECs (a more aggressive subtype) share similar molecular characteristics and have poor outcomes [14, 15]. Thus, the identification of the common molecular mechanisms responsible for tumorigenesis and progression of these aggressive ECs would be useful in developing diagnostic and therapeutic strategies for improving diagnosis and patient survival.

Overexpressed miR-125b [16] could function as an oncogene, whereas downregulated miRNAs, including miR-194 [17], miR-130b [18], miR-106b [19] and miR-34 [20], could work as tumor suppressors in aggressive ECs. Previous studies have shown that miR-101 is downregulated in both endometrioid and serous EC tissues [20] and it inhibits serous EC cell proliferation [21]. Although most evidence indicates the tumor suppressor activity of miR-101 in cancer cells, conflicting evidences also indicate that miR-101 can act as an oncogenic miRNA in other malignancies [22, 23, 24, 25], consistent with the notion that a miRNA may exhibit diverse context-dependent functions through distinct pathways [26]. Currently, little is known about the biological function of miR-101 and its actual targets in the aggressive type of EC.

Here, we report that miR-101 can suppress proliferation, the epithelial-mesenchymal transition (EMT)-associated migration and invasion, and stem cell-like phenotype of aggressive EC cells, at least through targeting EZH2, MCL-1 and FOS. Furthermore, we found that decreased miR-101 expression correlates inversely with increased *EZH2, MCL-1* and *FOS* expression in EC tissues. Our results suggest that miR-101 exerts its novel tumor suppressive activities in aggressive ECs by modulating multiple critical oncogenes.

## RESULTS

### MiR-101 is downregulated in aggressive EC cell lines and modulates cell proliferation

**Figure 1:**
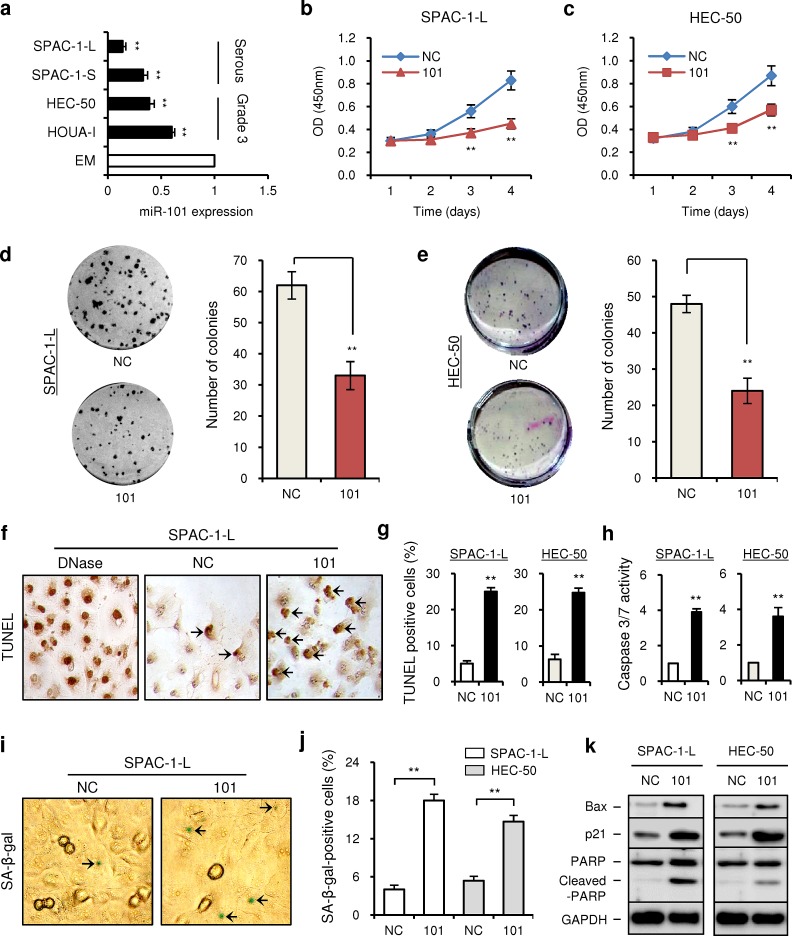
MiR-101 is downregulated in aggressive EC cell lines and modulates cell proliferation (a) Relative miR-101 expression of four aggressive endometrial cancer cell lines and immortalized endometrial epithelial cell line EM were examined with the quantitative real-time RT-PCR (qRT-PCR) assay. The expression of GAPDH was used as a normalization control, and the results are presented as the fold-change in expression compared with EM. Effects of ectopic expression of miR-101 on the proliferation of SPAC-1-L cells (b) and HEC-50 cells (c) were assessed with cell counting kit-8 assay. Clone formation assays were performed in SPAC-1-L (d) and HEC-50 (e) cells transduced with pre-miR-101 (101) or pre-miRNA negative control (NC). (f) Representative images of TUNEL assay in SPAC-1-L cells at 72 hours after transfection. Arrows indicate TUNEL-positive cells. (g) The percentages of TUNEL-positive SPAC-1-L and HEC-50 cells. (h) SPAC-1-L and HEC-50 cells were transfected with 101 or NC for 72 hours, and the relative ratio of caspase-3/7 activities were determined. (i) SA-β-gal staining analysis in SPAC-1-L cells transfected with 101 or NC at 72 hours after transfection. Arrows indicate blue senescent cells positive for SA-β-gal staining. (j) The percentages of SA-β-gal-positive SPAC-1-L and HEC-50 cells. (k) Western blot analysis of p21, Bax, total PARP and cleaved PARP in SPAC-1-L and HEC-50 cells after transduction with 101 or NC. ^**^P < 0.01.

To investigate the role of miR-101 in EC cells, we first measured the endogenous miR-101 expression level in four aggressive EC cell lines (serous: SPAC-1-L and S; poorly-differentiated endometrioid: HEC-50 and HOUA-I), compared to that of the immortalized human endometrial epithelial cell EM. Quantitative analysis (qRT-PCR) demonstrated that miR-101 expression was downregulated in all 4 EC cell lines. The greatest reduction of miR-101 levels was found in highly invasive SPAC-1-L and S cells (Figure 1a), indicating that miR-101 might be a tumor suppressor in aggressive subtype of EC.

To assess the biological role of miR-101, we evaluated the effects of miR-101 on EC cell proliferation. MiR-101 levels could be elevated in the pre-miR-101 (101)-transfected SPAC-1-L (7-fold) and HEC-50 (6-fold) cells compared with pre-miRNA negative control (NC)-transfected cells (Additional file 1: Figure S1a). Re-expression of miR-101 in these cells led to decreased cell proliferation at 72 and 96 hours post-transfection, as measured by cell counting kit-8 assays (Figure 1b and C). To evaluate a longer-term impact, we performed colony formation assays on SPAC-1-L and HEC-50 cells transfected with 101 or NC. As expected, overexpression of miR-101 significantly decreased the clonogenic ability of both cells (Figure 1d and e).

To determine whether the reduction of cell proliferation following miR-101 treatment was due to the induction of apoptosis, we examined the nuclear DNA fragments that resulted from apoptosis using a colorimetric TUNEL staining assay. Positive-control, DNase-treated SPAC-1-L cells exhibited the expected intense TUNEL labeling, and the percentages of apoptotic cells with brown stained nuclei were significantly higher in 101-transfected SPAC-1-L and HEC-50 cells compared with their controls (Figure 1f and g). In accordance with these results, caspase-3/7 activity was increased in response to 101 compared with NC (Figure 1h). To gain further insight into the anti-proliferative effect of miR-101, we next evaluated whether the decreased proliferation upon miR-101 overexpression was a result of cellular senescence. SPAC-1-L and HEC-50 cells transfected with 101 or NC were subsequently subjected to senescence-associated β-galactosidase (SA-β-gal) staining and morphology analysis 3 days after transfection. Introduction of miR-101 in SPAC-1-L and HEC-50 cells caused senescence-like phenotypes, such as positive staining for SA-β-gal (Figure 1i and j) and enlarged, flattened cell morphology (Additional file 1: Figure S1b). Furthermore, immunoblot analysis revealed that miR-101 overexpression markedly enhanced the expression of pro-apoptotic gene Bax, apoptosis marker cleaved-PARP and senescence marker p21 in either cell line (Figure 1k). These results suggest that miR-101 can trigger apoptosis and/or senescence programs and in turn suppress the proliferative capacity of aggressive EC cells.

### MiR-101 inhibits aggressive EC cell migration, invasion and EMT

**Figure 2:**
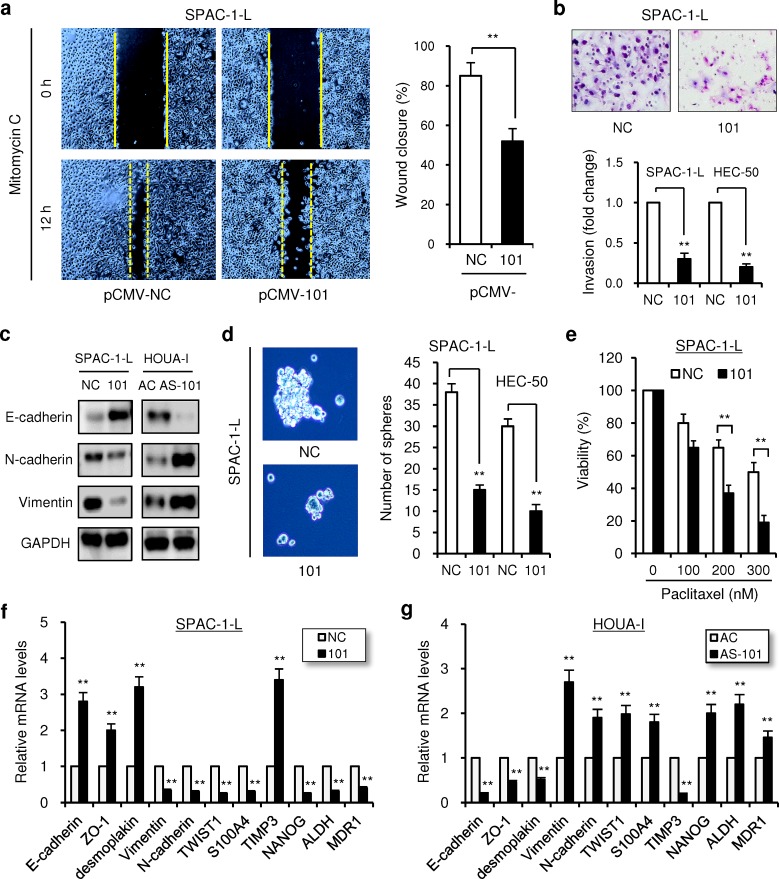
Overexpression of miR-101 inhibits aggressive EC cell migration, invasion and EMT (a) The scratch wound healing assay was performed in SPAC-1-L cells transfected with either 101 or NC in the presence of Mitomycin C. The wound healing was determined at the time points as indicated. Solid lines: the initial wound boundaries; dashed lines: the boundaries of migrated cells. Bars represented the percentage of wound healing. (b) SPAC-1-L and HEC-50 cells were transfected with 101 or NC, and subjected to invasion assay. Photos were representative fields of invasive cells on the membrane. (c) Western blot analysis of E-cadherin, N-cadherin and Vimentin was performed in SPAC-1-L and HOUA-I cells at 72 hours after 101 or anti-miR-101 inhibitor (AS-101) transfection, respectively. (d) Representative images (left) and quantification (right) of sphere formation in SPAC-1-L and HEC-50 cells transfected as indicated. (e) SPAC-1-L cells transfected with 101 or NC were treated with Paclitaxel for 24 hours. Cell viability was measured with cell counting kit-8 assay. The values were expressed as the percentage of viable cells, with the viability of DMSO-treated cells set at 100%. Relative mRNA expression of EMT, invasion and stemness-related genes (normalized to GAPDH) in SPAC-1-L cells after miR-101 overexpression (f), or in HOUA-1 cells following miR-101 knockdown (g), were determined using *qRT-PCRs*. Anti-miRNA negative control (AC). ^**^P < 0.01.

We evaluated the effects of miR-101 on cell migration and invasion of SPAC-1-L and HEC-50 cells with relatively lower levels of miR-101, or on HOUA-I cells, which expresses relatively higher levels of miR-101. Stable SPAC-1-L cell lines overexpressing miR-101 were established by transfection of miR-101 expression vector (pCMV-101), and the miRNA levels were analyzed using qRT-PCR (Additional file 2: Figure S2a). We first examined the effect of miR-101 stable overexpression on SPAC-1-L cell migration using wound healing assay in the presence of proliferation inhibitor Mitomycin C. pCMV-101-overexpressing SPAC-1-L cells had slower motility (wound closure) compared with pCMV-control vector (pCMV-NC)-transfected cells (Figure 2a). Furthermore, we investigated whether suppression of miR-101 expression would induce EC cell migration. A miR-101-specific inhibitor (AS-101) was introduced into HOUA-I cells to neutralize endogenous miR-101 activity (Additional file 2: Figure S2b). The inhibition of miR-101 by AS-101, but not anti-miRNA negative control (AC), significantly increased migratory abilities in a transwell chamber assays (Additional file 2: Figure S2c). Matrigel invasion assay showed that transient transfection of 101 dramatically reduced the invasion of SPAC-1-L and HEC-50 cells (Figure 2b). Although SPAC-1-L cells are highly invasive, this cell displays an epithelial-like morphology, thus we next examined whether the silencing of miR-101 expression can cause a gain in mesenchymal features. The inhibition of miR-101 by AS-101 in SPAC-1-L cells changed cell morphology from an epithelial to a more mesenchymal phenotype and increased cell invasion (Additional file 2: Figure S2d, e and f). Consistent with these observations, knockdown of miR-101 by AS-101 in HOUA-I cells induced cell scattering and invasiveness in Matrigel (Additional file 2: Figure S2g and h). Western blot analyses showed that re-expression of 101 in SPAC-1-L cells was associated with upregulation of epithelial marker E-cadherin and downregulation of mesenchymal markers N-cadherin and Vimentin. Along similar lines, loss of miR-101 expression in HOUA-I cells led to marked reduction of E-cadherin and increased expression of N-cadherin and Vimentin (Figure 2c). These data suggest that ectopic expression of miR-101 can reduce mesenchymal phenotype and invasive ability of aggressive EC cells.

Tumor cells undergoing epithelial-mesenchymal transition (EMT) often acquire CSC-like characteristics, including enhanced ability to form mammospheres, drug resistance and upregulation of stem cell markers [27, 28]. Therefore we tested whether miR-101 overexpression can reduce CSC properties. We found that 101-transfected SPAC-1-L and HEC-50 cells formed significantly smaller numbers of spheres in serum-free suspension cultures (Figure 2d). In addition, upregulation of miR-101 sensitized SPAC-1-L cells to paclitaxel toxicity, as measured by cell counting kit-8 assay (Figure 2e). In contrast, silencing of miR-101 in HOUA-I cells decreased apoptotic response to paclitaxel (Additional file 2: Figure S2i). Of importance, overexpression of 101 in SPAC-1-L cells increased the mRNA levels of epithelial markers (*E-cadherin*, *ZO-1* and *desmoplakin*) and metastasis suppressor gene *TIMP-3*, however decreased the mRNA levels of mesenchymal markers (*N-cadherin* and *Vimentin*), known EMT inducer *TWIST1*, metastasis promoter *S100A4*, CSC markers (*ALDH* and *NANOG*) and chemoresistance marker *MDR1* (Figure 2f). These changes were reversed by knockdown of miR-101 in HOUA-I cells (Figure 2g). Taken together, these findings suggest that miR-101 suppresses the EMT-associated phenotypes of aggressive EC cells.

### MiR-101 directly targets *EZH2, MCL-1* and *FOS*

**Figure 3:**
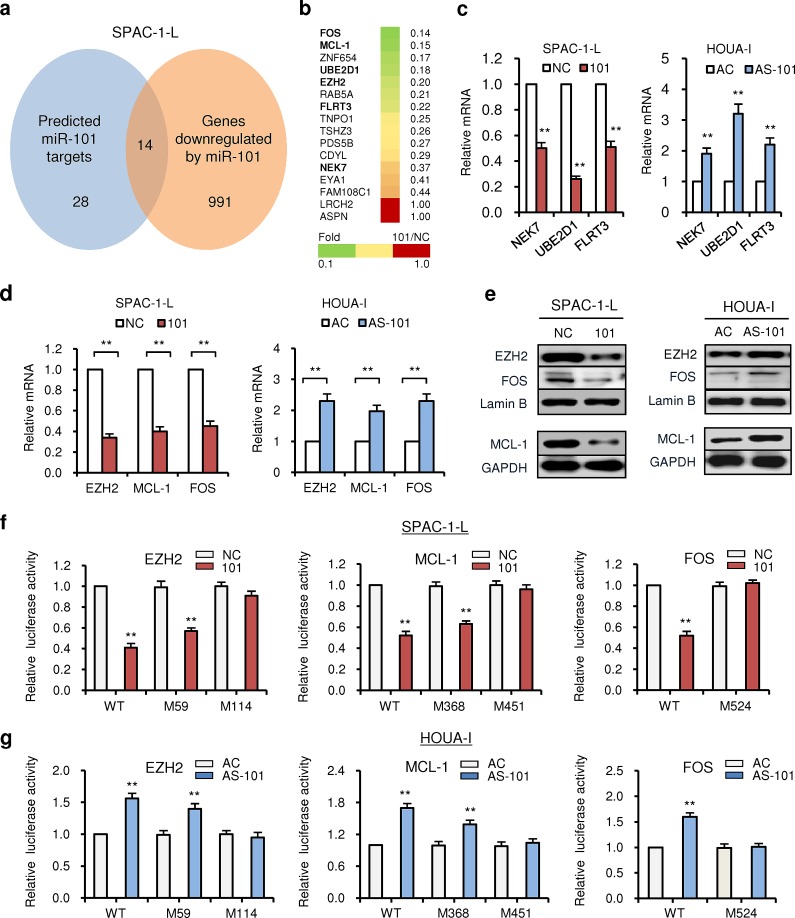
MiR-101 directly targets *EZH2, MCL-1* and *FOS* (a) Total RNAs of SPAC-1-L cells transduced with 101 or NC were used for a microarray analysis. Predicted 28 potential targets of miR-101 were shown (left). Transcripts of 991 genes were found to be downregulated by miR-101 mimic (right). The overlapping 14 genes were determined. (b) The heatmap (green and yellow: downregulated 14 genes by miR-101; red: two predicted miR-101 target genes *ASPN* and *LRCH2* whose expression was not affected by transfection with 101). The numbers indicated the fold change of gene expression (Fold 101/NC) in SPAC-1-L cells transduced with 101 compared to NC, as determined by microarray. (c) qRT-PCRs showed the downregulation of *NEK7*, *UBE2D1* and *FLRT3* after miR-101 overexpression in SPAC-1-L cells (left), and the upregulation of these genes after miR-101 knockdown in HOUA-I cells (right). (d) qRT-PCRs showed the downregulation of *EZH2*, *MCL-1* and FOS after miR-101 overexpression in SPAC-1-L cells (left), and the upregulation of these genes after miR-101 knockdown in HOUA-I cells (right). (e) Western blotting for EZH2, MCL-1 and FOS after transfection with 101 in SPAC-1-L cells (left), or with AS-101 in HOUA-I cells (right). Luciferase reporter assays confirmed that *EZH2*, *MCL-1* and *FOS* were direct targets of miR-101 in SPAC-1-L (f) and HOUA-I cells (g). WT: Wild-type; M: Mutant 3'-UTR. ^**^P < 0.01.

In order to identify potential targets of miR-101, we combined *in silico* analysis and microarray gene expression analysis using 101-transfected SPAC-1-L cells relative to NC-transduced control cells. First, we identified 28 genes predicted to be targeted by miR-101 (Additional file 3: Figure S3a) by using three prediction softwares (TargetScan, miRNA.org and DIANA-microT) with high precision and sensitivity [29, 30]. We further detected 991 gene transcripts downregulated (*P* < 0.05) by miR-101 overexpression in SPAC-1-L cells using genome-wide arrays, and cross-referenced these genes with 28 predicted genes to determine the overlapping 14 genes (Figure 3a and b). Among them, we again identified three cancer-related genes (*NEK7*, *UBE2D1* and *FLRT3*) (Figure 3b), whose mRNA expressions were repressed by miR-101 overexpression in SPAC-1-L cells and induced by AS-101 in HOUA-I cells (Figure 3c). Interestingly, we noticed that a group of genes (*EZH2*, *MCL-1* and *FOS*) containing putative binding site(s) on their 3'-untranslated regions (3'-UTRs) (Additional file 3: Figure S3b) were also markedly downregulated (at least 2-fold by microarray analysis) in 101-transduced SPAC-1-L cells (Figure 3b), and fell into known miR-101 targets in human diseases except EC (Additional file 3: Figure S3c). In agreement with this, a negative correlation between endogenous miR-101 levels and *EZH2*, *MCL-1* and *FOS* mRNA expression was found in EC cells (Figure 1a and Additional file 3: Figure S3d). Next, we confirmed that endogenous mRNA and protein levels of these genes were downregulated in 101-transfected SPAC-1-L cells (Figure 3d and 3e, left panel). Knockdown of miR-101 by AS-101 conversely induced the mRNA and protein levels of EZH2, MCL-1 and FOS in HOUA-I cells (Figure 3d and 3e, right panel). Luciferase reporter assay showed that exogenous miR-101 in SPAC-1-L cells exerted repressive effects on luciferase activity of wild-type *EZH2*, *MCL-1* and *FOS* 3'-UTR vectors (Figure 3f), and administration of AS-101 into HOUAI-I cells resulted in an increased luciferase activity (Figure 3g). When mutations were introduced into the potential 3'-UTR miR-101 binding sites, only mutation at position 114-21 for *EZH2*, position 451-8 for *MCL-1* and position 524-31 for *FOS* 3'-UTR prevented the downregulation of reporter activities by miR-101 (Figure 3f). These results were also confirmed in HOUA-I cells (Figure 3g), verifying that the suppressive effects of miR-101 is mainly due to direct interaction with binding sites in the *EZH2*, *MCL-1* and *FOS* 3'-UTR at these perfect 8-mer sites (Additional file 3: Figure S3b).

### Knockdown of EZH2, MCL-1 and FOS expression repress proliferation, invasion and CSC-like phenotypes of aggressive EC cells

**Figure 4:**
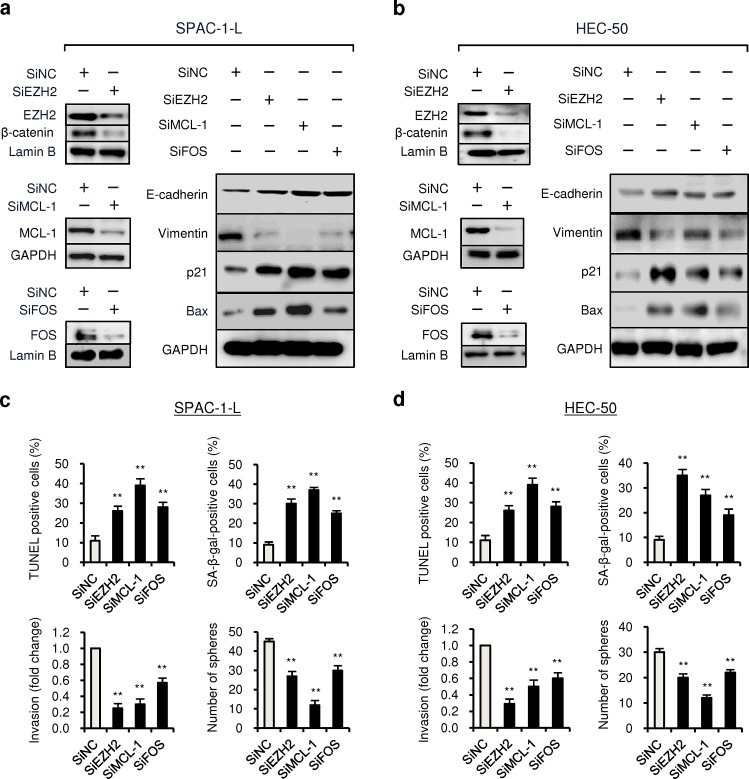
Knockdown of EZH2, MCL-1 and FOS expression reduce proliferation, invasion and CSC-like phenotypes of aggressive EC cells SPAC-1-L and HEC-50 cells were transduced with specific siRNAs (siEZH2, siMCL-1, siFOS) or control siRNA (siNC), and western blotting of EZH2, MCL-1 and FOS expression (a and b) along with analysis of cell apoptosis, senescence, invasion and sphere formation ability (c and d) were conducted as indicated. ^**^P < 0.01.

To evaluate whether suppression of EZH2, MCL-1 and FOS is responsible for the miR-101-mediated tumor suppression, we knocked down these three genes using specific siRNAs in SPAC-1-L and HEC-50 cells, and found that decreased expression of these genes (Figure 4a and b) was able to significantly induce cell apoptosis and senescence (Figure 4c and d, upper panel), possibly accounting for the inhibitory effects of EZH2, MCL-1 and FOS siRNA on EC cell proliferation (Additional file 4: Figure S4). We also observed that transfection EZH2, MCL-1 and FOS siRNA significantly inhibited the ability of SPAC-1-L and HEC-50 cells to invade and to form spheres (Figure 4c and d, lower panel), mimicking the tumor suppressive effects upon miR-101 overexpression. We found that the protein expression of epithelial marker E-cadherin, senescence marker p21, pro-apoptosis factor Bax were markedly upregulated, and mesenchymal marker Vimentin and nuclear β-catenin levels (a stem cell-related gene) were greatly downregulated in both cell lines, following the transfection with EZH2, MCL-1 and FOS siRNA (Figure 4a and b), suggesting that suppression of multiple oncogenes including *EZH2*, *MCL-1* and *FOS* by miR-101 attenuates cell proliferation and invasiveness, and abrogates CSC-like properties in aggressive EC cells.

### MiR-101 and its targets show inverse expression levels and in EC tissues

**Figure 5:**
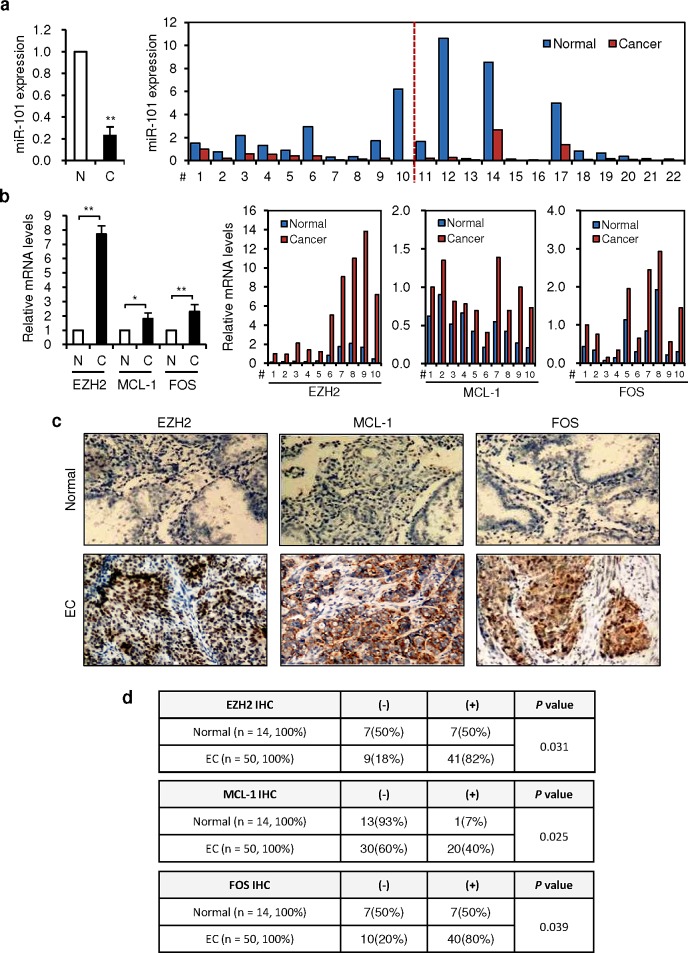
An inverse correlation between decreased miR-101 expression and elevated expressions of EZH2, MCL-1 and FOS in ECs (a) qRT-PCR analysis showed the downregulation of miR-101 in 22 pairs (#1-22) of EC tissues (C) and their adjacent normal tissues (N), depicted as normalized data (left, fold changes) and raw expression values (right, calculated with the 2^−ΔΔCt^ method). (b) Expression analysis (qRT-PCR) of *EZH2*, *MCL-1* and *FOS* in 10 pairs of ECs (#1-10) compared with adjacent normal tissues, depicted as normalized data (left, fold changes) and raw expression values (right, calculated with the 2^−ΔΔCt^ method). (c) Immunochemical analysis (IHC) of EZH2, MCL-1 and FOS in a separate set of samples (50 patients with grade 3 endometrioid ECs and 14 normal tissues) revealed that abundant EZH2, MCL-1 and FOS expression in cancer cells, but not in benign cells. (d) High expression of EZH2, MCL-1 and FOS was found in ECs, as measured by the immunostaining scores. ^*^P < 0.05; ^**^P < 0.01.

Since miR-101 and its targets appear to play an important role in EC progression, we compared the endogenous expression levels of miR-101, *EZH2*, *MCL-1* and *FOS* between EC tissues and adjacent normal tissues by qRT-PCR analysis. As previously reported [20, 21], miR-101 levels were lower in 22 (#1-22) ECs than in normal tissues (Figure 5a). In contrast, among 10 tumors (#1-10) from the same set of samples, the mRNA expression of its targets (*EZH2*, *MCL-1* and *FOS*) was significantly higher in cancer tissues (Figure 5b). These results show an inverse association between loss of miR-101 and upregulation of *EZH2*, *MCL-1* and *FOS*. We also assessed EZH2, MCL-1 and FOS levels by immunochemical analysis (IHC) in a separate set of samples including 50 grade 3 endometrioid ECs and 14 benign endometrial samples (Figure 5c and d). When comparing normal samples, EC tissues showed elevated EZH2, MCL-1 and FOS expression (Figure 5, Table S2 and Table S3). These data are consistent with our *in vitro* evidences showing that miR-101 targets EZH2, MCL-1 and FOS in aggressive EC cells, and suggest that the miR-101-EZH2/MCL-1/FOS signaling axis serves as a novel mechanism underlying endometrial tumorigenesis and metastasis, providing potential new therapeutic targets.

## DISCUSSION

The miRNAs can simultaneously bind and silence multiple target genes, and indirectly regulate downstream pathways of these target genes, thus affecting malignant cellular behaviors [4, 5]. The clarification of functional roles and the underlying mechanisms of certain miRNAs that are aberrantly expressed in human tumors would provide valuable insight for the development of new miRNA-based therapies. Although previous findings support a tumor suppressor role of miR-101 in human malignancies, the overexpression of miR-101 has been also detected in other tumor tissues and miR-101 may exhibit its tumor-promoting properties in certain contexts [22-25]. The role and its novel downstream targets of miR-101 in aggressive endometrial cancer have not yet been described.

**Figure 6:**
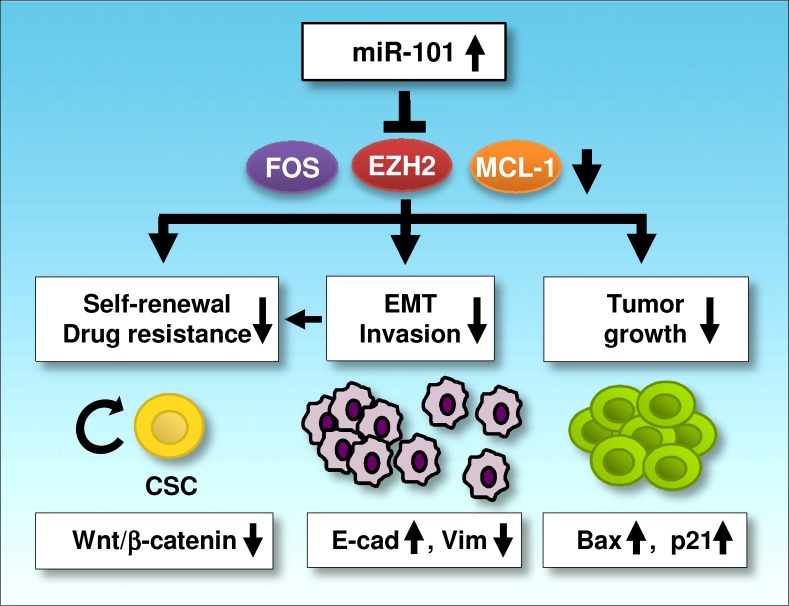
A schematic model depicting the mechanisms of miR-101-mediated tumor suppression In aggressive EC cells, miR-101 can downregulate three novel oncogenes (*EZH2*, *MCL-1* and *FOS*), in turn suppresses tumor cell proliferation, EMT, cell migration, invasion and cancer stemness, and enhances chemosensitivity to paclitaxel (E-cadherin: E-cad; Vimentin: Vim).

Here, we showed that miR-101 levels was significantly downregulated in aggressive EC cell lines compared with immortalized human endometrial epithelial cells. Increasing levels of miR-101 in invasive EC cells reduce cell proliferation, most likely due to miR-101-induced apoptosis and senescence. Moreover, upregulation of miR-101 in EC cells can remarkably reverse EMT-associated phenotypes, including reduced cell migration and invasiveness, restored sensitivity to Paclitaxel and impaired sphere formation. We further indentified potential miR-101 targets by computational prediction and microarray screening. Among them, three oncogenes (*EZH2*, *MCL-1* and *FOS*) were verified them as direct targets of miR-101. The silencing of these genes with specific siRNAs mimics the tumor suppressive effects of miR-101 overexpression in EC cells. Further qRT-PCR and IHC experiments clearly reveal that lower miR-101 expression is inversely correlated with higher EZH2/MCL-1/FOS protein levels in EC tissues. Our results provide the first evidence that, in aggressive EC cells, miR-101 governs multiple malignant phenotypes including proliferation, migration, invasion and cancer stemness, at least partly via downregulating the expression of *EZH2, MCL-1 and FOS* (Figure 6). Our data brings a new insight into the mechanisms by which loss of miR-101 expression promotes EC initiation and progression

EZH2 is a master regulatory gene that has a critical role in cancer development through its ability to epigenetically silence tumor suppressor genes [31]. High EZH2 expression serves as a marker of advanced or aggressive diseases in many human tumors [32]. Overexpression of EZH2 confers an invasive phenotype on cancer cells via downregulation of tumor suppressor TIMP-3 [33]. Knockdown of EZH2 expression in cancer cells induced cellular senescence, apoptosis and mesenchymal-to-epithelial transition features [34, 35]. EZH2 activity has been reported to be required for CSC maintenance [36]. Recent studies have linked EZH2 overexpression to EC invasion/metastasis [37, 38], and shRNA-mediated EZH2 inhibition in EC cells decreased proliferation, migration and invasion via either upregulation of E-cadherin or inactivation of Wnt/β-catenin signaling [39]. Although the interaction between miR-101 and EZH2 has been verified in other tumors [40, 41], our results suggest that miR-101 not only promotes cell apoptosis and senescence, but also suppresses the EMT and CSC properties of aggressive EC cancer cells, at least in part through attenuating EZH2 expression, with a consequential elevation of Bax, p21, epithelial markers and TIMP-3, downregulation of mesenchymal markers as well as suppression of Wnt/β-catenin signaling evidenced by decreased β-catenin levels in the nucleus (Figure 4a and b).

In addition to EZH2, two miR-101 targets (MCL-1 and FOS) tested in our present study, have been reported to regulate tumorigenesis and metastasis. Pro-survival protein MCL-1 is a crucial inhibitor of apoptosis [42] and senescence [43]. Increased MCL-1 levels are responsible for tumor cell proliferation [42], migration [44], self-renewal of CSC [45] and drug resistance to Paclitaxel [46]. Increased *MCL-1* mRNA expression has been found in EC tissues compared with matched normal tissues [47]. On the other hand, FOS was thought to enhance proliferation, motility and invasiveness of cancer cells [48, 49], and have oncogenic role in promoting the development of EMT [50]. The FOS levels were higher in EC than in normal endometrium [51]. A recent study reported that shRNA-mediated silencing of *FOS* in breast cancer cells upregulates Bax levels and induces apoptosis [52]. However, *FOS* may also have tumour-suppressor activity [53]. Thus, it is not clear whether the negative regulation of MCL-1 and FOS contributes to the suppression of EC cell proliferation, invasion and CSC-like phenotype by miR-101. Here, we showed that in aggressive EC cells, silencing of either *MCL-1* or *FOS* by specific siRNAs can induce cell apoptosis and senescence, inhibit EMT-associated cell invasion, and reduce sphere formation ability. Previous studies from hepatocellular carcinomas have suggested that MCL-1 [54] and FOS [49] were regulated by miR-101. In accordance with these findings, our study further confirmed that miR-101 directly targets *MCL-1* and *FOS* in aggressive EC cells, thus miR-101 may suppress multiple steps of EC metastasis through its combined effects on EZH2, MCL-1 and FOS.

In conclusion, we demonstrate that miR-101 suppresses cancer cell growth, invasiveness and self-renewal by simultaneously downregulating multiple oncogenes in aggressive ECs. The miR-101-EZH2/MCL-1/FOS axis serves as a novel mechanism underlying the invasive and stemness properties of EC cells. Therefore, the restoration of miR-101, or in combination with EZH2, MCL1 and FOS inhibitors, might be a potential therapeutic strategy for blocking EC initiation, progression and drug resistance.

## MATERIALS AND METHODS

### Cell lines and transient transfection

Human serous EC cell lines SPAC-1-L and SPAC-1-S were kindly provided by Dr. Fumihiko Suzuki(Tohoku University, Sendai, Japan), and maintained in RPMI-1640 medium (Sigma-Aldrich, St. Louis, MO) supplemented with 10% fetal bovine serum (FBS). The HEC-50 (JCRB Cell Bank, Osaka, Japan) and HOUA-I (RIKEN cell bank, Tsukuba, Japan) cell lines were derived from poorly-differentiated endometrioid EC, and grown in DMEM/F12 medium (Sigma-Aldrich, St. Louis, MO, USA) supplemented with 10% FBS. The immortalized human endometrial epithelial cell line EM [55] was a kind gift from Professor Satoru Kyo (Shimane University, Japan) and maintained in DMEM/F12 medium supplemented with 15% FBS. The cells were transfected using Lipofectamine 2000 (Invitrogen, Carlsbad, CA) transfection protocol, according to the manufacturer's instructions. 101, NC, AS-101, AC, siRNA-EZH2 (siEZH2), -MCL-1 (siMCL-1), -FOS (siFOS) and siRNA negative control (siNC) were obtained from Ambion (Austin, TX). Cells were transfected at final miRNA concentration of 40 nM, and final siRNA concentration of 10 nM, respectively.

### Plasmid and stable transfection

The pCMV-miR-101-3p (pCMV-101) vector carrying pre-microRNA-101-3p and the control vector (pCMV-NC) (OriGene, Rockville, MD) were transfected into SPAC1-L cells by Lipofectamine PLUS Reagent (Invitrogen, Carlsbad, CA) as described previously [56]. Transfected cells were selected using 400 μg/ml of G418 (Sigma-Aldrich, St. Louis, MO). Single cell colonies were selected six weeks following transfection.

### Quantitative real time RT-PCR (qRT-PCR) analysis of miRNA and mRNAs

Total RNA was extracted from cell lines with TRIzol reagents (Invitrogen, Carlsbad, CA). QRT-PCR was performed to quantify mature miRNA expression with the NCode miRNA qRT-PCR analysis (Invitrogen, Carlsbad, CA), or mRNA expression with the Takara SYBR Premix Ex Taq II (Takara, Japan). The forward primer for miR-101-3p was obtained from Invitrogen (5'-CCGGTACAGTACTGTGATAACTGAA-3'). GAPDH or control miRNA U6 was used for normalization. The primers used for mRNA expression were obtained from the PrimerBank database (http://pga.mgh.harvard.edu/primerbank/). QRT-PCR was performed with the Applied Biosystems 7300 real-time PCR system (Applied Biosystems).

### Cell viability assay, cell proliferation assay and colony formation assay

Cells (5 × 10^3^) were plated in 96-well plates for 24 hours and then treated with DMSO or varying doses of paclitaxel (Cell Signaling Technology, Beverly, MA) as indicated. Cell viability was determined by the Cell Counting Kit-8 (Dojindo, Kumamoto, Japan) 24 hours later. The absorbance was determined at 450 nm using a microplate reader, and the percentage absorbance was calculated against DMSO-treated cells. For cell proliferation assay, cells (2.5 × 10^3^) were plated in 96-well plates, transfected with 101or NC as described above, and the growth curve of cells, covering a total of four days of culturing, were plotted with the Cell Counting Kit-8 method. For colony formation assay, approximately 500 cells were added to each well of a 6-well culture plate, and each experiment was performed in triplicate. After 12 days of culture at 37°C, cells were fixed with 10% formalin and stained with 10% Giemsa solution. The number of colonies containing ≥ 50 cells was counted under a microscope.

### Cell migration, invasion assay and wound healing assay

Invasion assays were performed were performed using Transwell system (8-μm pore size, matrigel-coated polycarbonate membrane BD Biosciences, Bedford, MA), as described previously [57]. Briefly, 5 × 10^4^ cells resuspended in serum-free medium was added to the upper inserts. In the lower chamber, 750 μl medium supplemented with 10% FBS served as a chemoattractant. After incubation for 24 hours, the number of cells adhering to the lower surface of membrane was counted under the microscope. Migration assays were carried out in the same way as the invasion assay, except that the membrane was not coated with matrigel, and the incubation time was 12 hours. Wound healing assay was also used to assess cell migration as previously reported [58]. Confluent cells were scraped by 200-μl pipette tip to create an artificial wound, and incubated with fresh medium containing Mitomycin C (5 μg/ml) for 12 hours. Distance migrated was quantitated by taking pictures at 0 and 12 hours.

### Apoptosis assay (TUNEL staining)

Apoptosis was determined using the terminal deoxynucleotidyl transferase mediated dUTP nick-end labeling (TUNEL) assay (DeadEnd Colorimetric TUNEL System, Promega, Madison, WI), following the manufacturer's instructions. Cells were transfected with as described above. After 72 hours, cells were fixed, treated with proteinase K followed by the incubation with the TdT reaction mixture. Cells were then incubated in streptavidin-HRP, and the color was developed with diaminobenzidine as a chromogen. Apoptotic nuclei exhibiting fragmented DNA were stained dark brown and the number of apoptotic cells was expressed as a percentage of the total population in four separate 200× fields. Cells exposed to RQ1 DNase solution (Promega, Madison, Wisconsin) served as a positive control.

### Caspase-Glo 3/7 assay

3 × 10^3^ cells were plated in triplicates in 96-well plates and transfected as indicated. The enzymatic activity of Caspase-3 and 7 was evaluated as an indicator of apoptosis using a Caspase-Glo 3/7 assay kit according to the manufacturer's instructions (Promega, Madison, WI). Luminescence was measured after 3 hours of incubation with the caspase substrate.

### Senescence assay (SA-β-gal staining)

The activity of senescence-associated β-galactosidase (SA-β-gal), a marker of cellular senescence, was determined by using the Cellular Senescence Assay Kit (Chemicon International, Temecula, CA, USA) according to the manufacturer's instructions. 72 hours after transfection as indicated above, cells were washed twice with PBS, fixed with 1X fixing solution and incubated at room temperature for 10 minutes. After removing the fixing solution, cells were washed twice again with PBS, and incubated overnight with freshly prepared 1X SA-β-gal detection solution at 37°C, without CO2 and protected from light. The percentages of blue-stained senescent cells (SA-β-gal–positive) were determined by counting 150 to 200 cells in six microscopic fields.

### Western blot analysis

Whole cell lysates were obtained using the M-Per Mammalian Protein Extraction Reagent (Pierce Biotechnology, Woburn, MA). Nuclear protein extracts were prepared using a Nuclear Extraction Kit (Chemicon International, Temecula, CA) according to the manufacturer's instructions. Total proteins (40 μg) and nuclear proteins (10 μg) were separated on 10% SDS-PAGE and transferred to nitrocellulose membranes. Antigen-antibody complexes were detected using the enhanced chemiluminescence (ECL) blotting analysis system (Amersham Pharmacia Biotech, Buckinghamshire, UK). The following antibodies were used for analysis: anti-EZH2 (5246, Cell Signaling, Danvers, MA), anti-PARP (9542, Cell Signaling, Danvers, MA), rabbit polyclonal anti-E-cadherin (A01589, GenScript, Edison, NJ), mouse monoclonal anti-N-cadherin (BD, Transduction, San Jose, CA), rabbit polyclonal anti-Vimentin (A01189, GenScript, Edison, NJ). Anti-MCL-1 (sc-819), anti-FOS (sc-52), anti-p21 (sc-397), anti-Bax (sc-493), anti-β-catenin (sc-1496), and anti-GAPDH (sc-47724) and anti-lamin B1 (sc-20682) were purchased from Santa Cruz Biotechnology (Santa Cruz Biotechnology, Santa Cruz, CA). GAPDH (whole cell lysate) and lamin B (nuclear protein) were blotted to show equal protein loading, respectively.

### Sphere formation assay

Spheres culture was performed as described [18]. Cells (1000 cells/ml) were grown in serum-free medium supplemented with N2 plus media supplement (Invitrogen, CA), 20 ng/ml epidermal growth factor (EGF), 20 ng/ml basic fibroblast growth factor (bFGF) (Invitrogen, CA), and 4 mg/ml heparin (Sigma-Aldrich, UK) for 14 days. Then the number of spheres larger than 50 μm was counted.

### Microarray analysis

SPAC-1-L cells were transfected with pre-101 or NC, and 72 hours after transfection total RNAs were isolated with TRIzol reagents (Invitrogen, Carlsbad, CA). Genome-wide gene expression analysis was performed using Superprint G3 Human GE 8×60k Microarray (Agilent Technologies) as previously described [59].

### Luciferase activity assay

The 3'-UTR vectors of human EZH2 and FOS were purchased from OriGene Technologies (Rockville, MD). Human MCL-1 3'-UTR cloned downstream of a firefly luciferase gene was obtained from Switchgear Genomics (Menlo Park, CA). Mutant constructs containing point mutations in the miR-101-binding sites were created using a quick-change site-directed mutagenesis kit (Stratagene, CA). Cells were seeded in 24-well plates and transfected with wild-type or mutated reporter vectors, or 40 nM of 101, AS-101 or their negative controls. Cells were collected 24 hours after transfection, and the luciferase activity was measured using the dual-luciferase reporter assay system (Promega, WI). The effects of miR-101 on MCL-1 3'-UTR was examined with the LightSwitch Luciferase Assay Reagent (Menlo Park, CA).

### Patients, tissue samples and immunohistochemistry analysis (IHC)

Formalin-fixed, paraffin-embedded blocks from 50 patients who underwent surgical resection of grade 3 endometrioid ECs at the Cancer Center, Sun Yat-Sen University, were analyzed for the expression of miR-101, EZH2, MCL-1 and FOS. The clinical and pathological characteristics of these patients were described in Table S1. 14 normal endometrium specimens served as normal comparators. The study has received Research Ethics Committee approval from the Sun Yat-Sen University. In addition, 22 pairs of endometrioid ECs and adjacent normal endometrial tissues were obtained for RNA extraction. The streptavidin-biotin peroxidase complex technique was used for staining as previously reported [28]. The primary antibodies against EZH2 (5246), MCL-1 (sc-819), FOS (sc-52), estrogen receptor(H-150) and progesterone receptor (SP2) were obtained from Cell Signaling (Beverly, MA), Santa Cruz Biotechnology (Santa Cruz, CA) and Lab Vision Corporation (Fremont, CA), respectively. PBS with without either primary or secondary antibody was applied as the negative control. Expression of EZH2, MCL-1 and FOS were scored according to the average staining intensity (SI) and the percentage of positive cells (PT) [28]. The SI score ranged from 0 (absent), 1 (weak), 2 (moderate) to 3 (strong). The PT score varied from 0 (0-5%), 1 (5-25%), 2 (26-50%), 3 (51-75%) to 4 (76-100%). The immunostaining score (IRS) was calculated as the sum of SI and PT (range 0-7). The IRS scores of tumors were defined as the following rule: negative (score = 0-2) and positive (score = 3-7). Tumors with positive ER or PR nuclear staining in > 10% of tumor cells were defined as ER or PR positive.

### Statistical analysis

All experiments were performed in triplicate. For *in vitro* results, values represent mean ± s.e. and were analyzed by Student's t-test or Wilcoxon matched-pairs test, when appropriate. The Fisher's exact test was used to compare the categorical data. Significance was defined as *P* < 0.05.

## SUPPLEMENTARY MATERIAL FIGURES AND TABLES


