# Supplementary material for: MicroRNA-101 targets EZH2, MCL-1 and FOS to suppress proliferation, invasion and stem cell-like phenotype of aggressive endometrial cancer cells
Source: Oncotarget. 2014 Jul 2;5(15):6049–62. doi: 10.18632/oncotarget.2157 (PMC4171612; doi:10.18632/oncotarget.2157)
Supplement: Supplementary file 1 [file oncotarget-05-6049-s001.pdf]

MicroRNA-101 targets EZH2, MCL-1 and FOS to suppress proliferation, invasion and stem cell-like phenotype of aggressive endometrial cancer cells

Supplementary Material

a

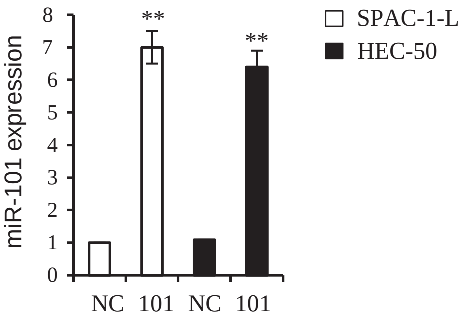

b

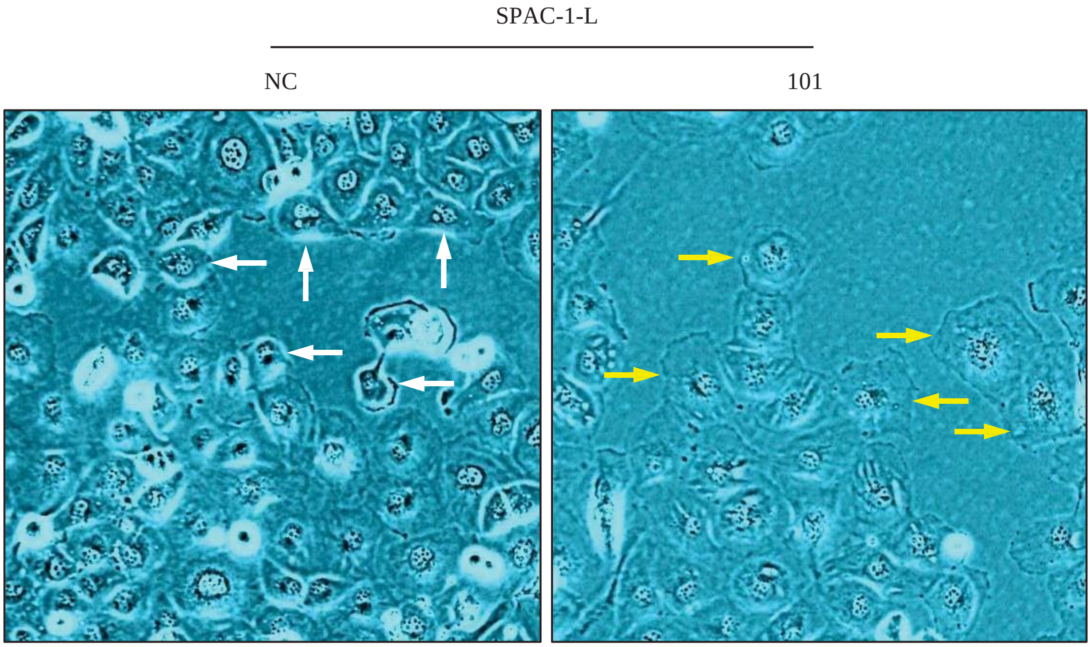

Figure S1: Upregulation of miR-101 promoted senescence-associated morphological changes in SPAC-1-L cells. (a) qRT-PCRs were conducted to quantify the relative miR-101 levels in SPAC-1-L and HEC-50 cells transfected with 101 or NC. $^{**}P < 0.01$. (b) SPAC-1-L cells transfected with miR-101 exhibited an enlarged and flattened morphology (yellow arrows) as compared to NC- transfected cells (white arrows).

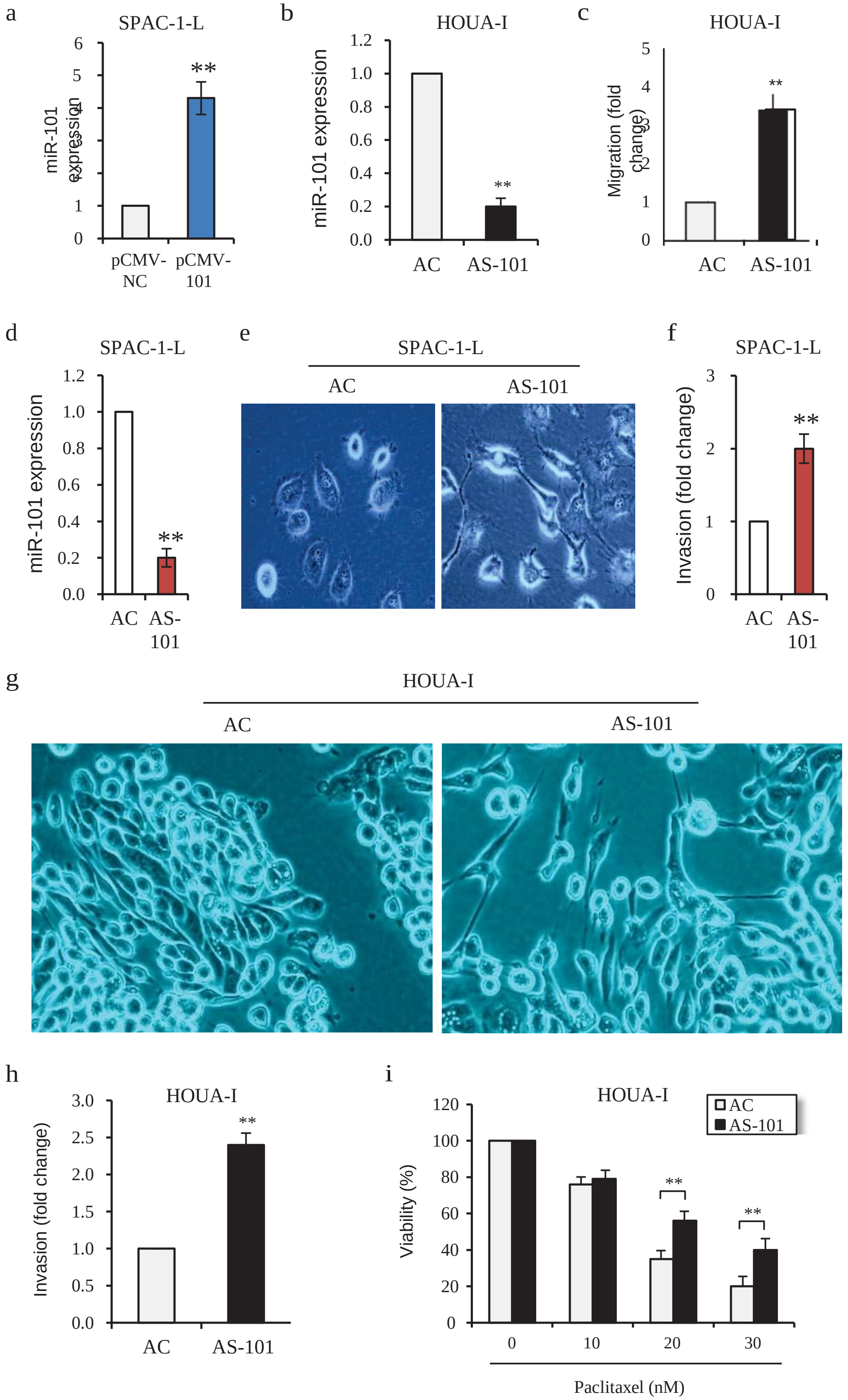

Figure S2: Knockdown of miR-101 induced the EMT-associated cell migration, invasion and drug resistance of EC cells. (a) qRT-PCR measurements of miR-101 levels in pre-101 (pCMV-101) or control vectors (pCMV-NC)-expressing SPAC-1-L cells. Knockdown of miR-101 in HOUA-I cells (b) induced cell migration in a transwell migration assays (c). SPAC-1-L cells transfected with AS-101 or AC (d) exhibited a more mesenchymal morphology (e) and enhanced invasive ability (f). Downregulation of miR-101 in HOUA-I cells was associated with EMT-like cell scattering (g) and increased invasion (h). (i) HOUA-I cells transfected with AS-101 or AC were treated with Paclitaxel for 24 hours. Cell viability was measured with cell counting kit-8 assay. The values were expressed as the percentage of viable cells, with the viability of DMSO-treated cells set at 100%. **P < 0.01.

a

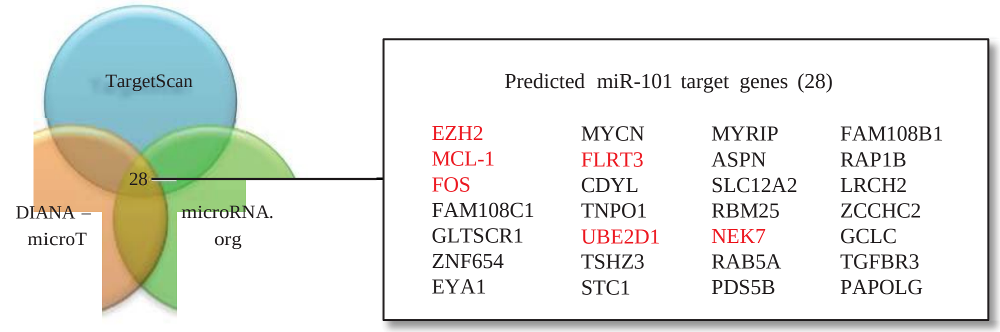

b

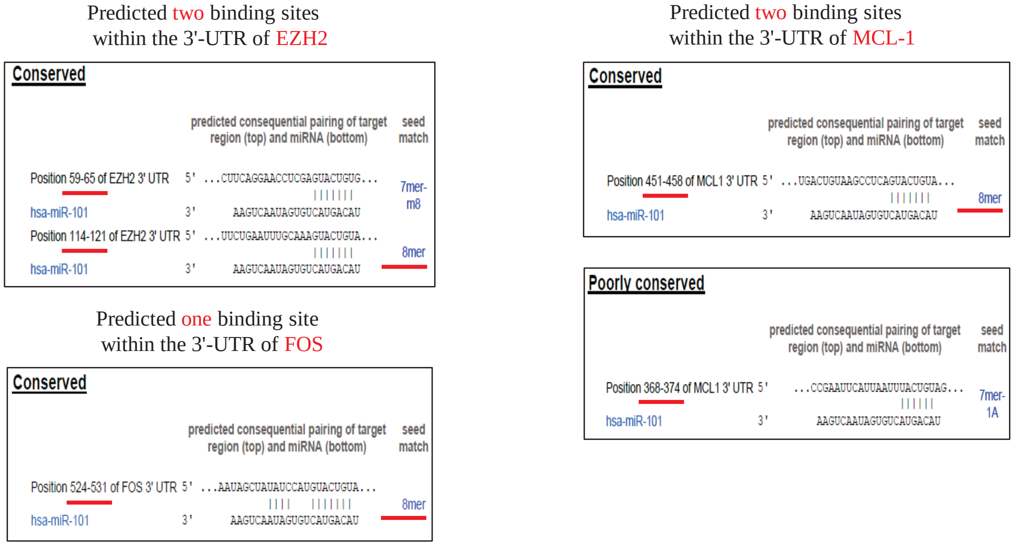

c

Reported miR-101 targets in human diseases	
Gene name	No. of Studies
EZH2	14
COX-2	4
MCL-1	3
FOS	2

d

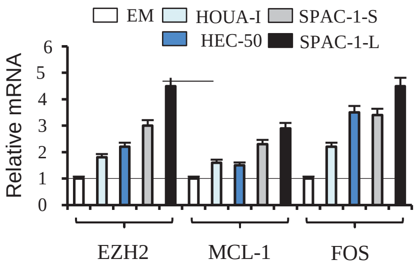

Figure S3: Candidate miR-101 target genes. (a) Venn diagrams showed 28 genes that were potential miR-101 targets predicted by TargetScan, miRNA.org and DIANA-microT. (b) Predicted (TargetScan) binding sites within the 3'-UTR of EZH2 (two binding sites), MCL-1 (two binding sites) and FOS (sole binding site). (c) EZH2, MCL-1 and FOS were known miR-101 targets in human diseases except endometrial cancer. (d) qRT-PCR analysis of EZH2, MCL-1 and FOS in four aggressive endometrial cancer cell lines and immortalized epithelial cell line EM.

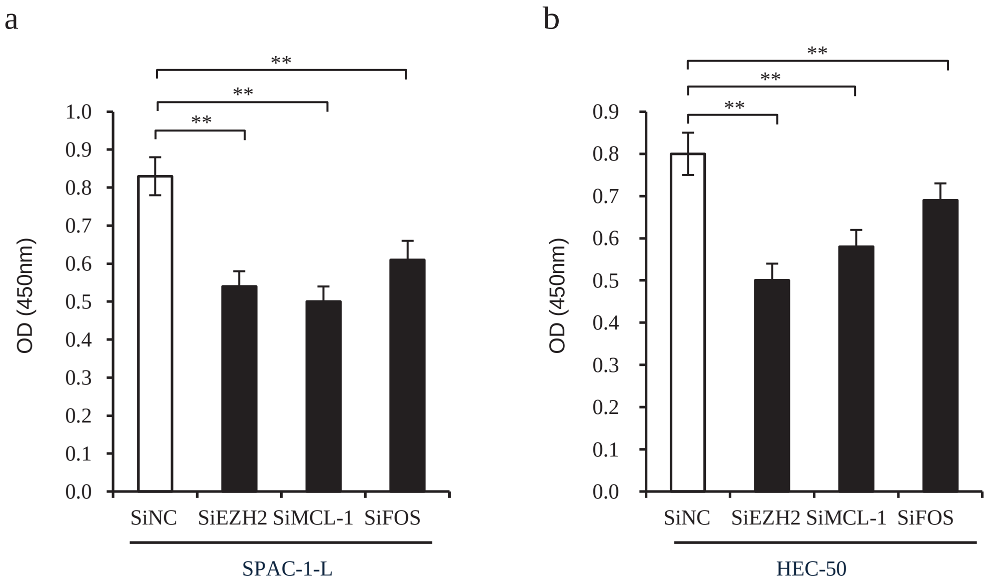

Figure S4: Specific siRNA-mediated downregulation of EZH2, MCL-1 and FOS suppressed proliferation in EC cells. Cell proliferation of SPAC-1-L (a) and HEC-50 (b) cells transfected with specific siRNAs (siEZH2, siMCL-1, siFOS) or control siRNA were quantified using cell counting kit-8 assays. $^{**}P < 0.01$.

Table S1: Clinical features of 50 endometrial cancer patients.

Characteristics	Factors	Number of cases	Percentage (%)
Age (years)	≤55	26	52
	> 55	24	48
FIGO Stage	I	28	56
	II	7	14
	III	12	24
	IV	3	6
Histology	endometriod	50	100
Histologic grade	I-II	0	0
	III	50	100
Deep Invasion	(-)	24	48
	(+)	26	52
Tumor diameter (cm)	≤3	21	42
	> 3	29	58
Nodal Matastasis	(-)	40	80
	(+)	10	20
Lymph vascular space invasion	(-)	39	78
	(+)	11	22
Ovarian Matastasis	(-)	45	90
	(+)	5	10
Estrogen receptor status	(-)	18	36
	(+)	32	64
Progesterone receptor status	(-)	14	28
	(+)	36	72

Table S2: IHC data on EZH2, MCL-1 and FOS expression in normal endometrium samples.

Case	Age(years)	Normal endometrium	EZH2 IHC	MCL-1 IHC	FOS IHC
1	36	yes	5	4	5
2	39	yes	5	0	5
3	43	yes	5	0	6
4	45	yes	4	0	5
5	44	yes	4	0	6
6	38	yes	3	0	0
7	37	yes	3	0	0
8	46	yes	2	0	4
9	35	yes	1	0	4
10	50	yes	1	0	0
11	49	yes	1	0	0
12	42	yes	1	1	1
13	41	yes	0	1	1
14	39	yes	0	1	1

IHC: immunochemical analysis of EZH2, MCL-1 and FOS, as measured by the immunostaining scores.

Table S3: IHC data on EZH2, MCL-1 and FOS expression in endometrial cancer samples.

Case	Age(years)	Tumor Type	FIGO Stage	Grade of tumor	EZH2 IHC	MCL-1 IHC	FOS IHC
1	34	EEC	1a	3	2	1	4
2	38	EEC	1b	3	7	5	4
3	40	EEC	1b	3	6	0	7
4	41	EEC	3c	3	4	1	2
5	42	EEC	2	3	5	1	2
6	43	EEC	3c	3	5	1	2
7	45	EEC	1a	3	6	0	7
8	46	EEC	1a	3	4	4	2
9	46	EEC	1a	3	2	1	1
10	47	EEC	3c	3	6	0	7
11	48	EEC	2	3	6	0	6
12	49	EEC	1a	3	6	0	7
13	49	EEC	2	3	7	4	1
14	49	EEC	3c	3	5	1	1
15	50	EEC	3c	3	5	1	5
16	50	EEC	1b	3	2	1	6
17	51	EEC	1b	3	7	4	5
18	51	EEC	4	3	7	6	5
19	53	EEC	1a	3	6	0	6
20	53	EEC	1b	3	6	0	7
21	53	EEC	1b	3	2	1	6
22	54	EEC	1a	3	1	5	6
23	54	EEC	1a	3	7	4	5
24	55	EEC	1a	3	7	0	7
25	55	EEC	1a	3	6	0	7
26	55	EEC	4	3	6	0	7
27	56	EEC	1a	3	2	4	6
28	57	EEC	1a	3	7	3	4
29	57	EEC	1a	3	6	2	6
30	57	EEC	3c	3	7	5	6
31	59	EEC	3c	3	5	1	1
32	59	EEC	4	3	5	1	2
33	59	EEC	1a	3	7	0	7
34	59	EEC	1a	3	6	2	6
35	60	EEC	3a	3	7	5	7

36	60	EEC	2	3	7	4	5
37	60	EEC	3a	3	2	2	1
38	61	EEC	1a	3	7	0	7
39	61	EEC	1b	3	7	5	7
40	61	EEC	2	3	6	0	6
41	62	EEC	1a	3	7	3	4
42	62	EEC	1a	3	7	0	7
43	64	EEC	2	3	7	5	7
44	64	EEC	3a	3	1	4	6
45	67	EEC	3c	3	7	6	5
46	68	EEC	3c	3	4	4	5
47	69	EEC	1a	3	7	4	5
48	72	EEC	2	3	6	0	6
49	75	EEC	1a	3	7	3	4
50	76	EEC	1b	3	2	1	6

EEC: endometrioid endometrial adenocarcinoma.
IHC: immunochemical analysis of EZH2, MCL-1 and FOS, as measured by the immunostaining scores.
